# Characterization of Human Balance through a Reinforcement Learning-based Muscle Controller

**DOI:** 10.1371/journal.pone.0320211

**Published:** 2025-04-01

**Authors:** Kübra Akbaş, Carlotta Mummolo, Xianlian Zhou

**Affiliations:** 1 Department of Biomedical Engineering, New Jersey Institute of Technology, Newark, New Jersey, United States of America; 2 Department of Mechanics, Mathematics, and Management at Politecnico di Bari, Bari, Italy; Universita Politecnica delle Marche Facolta di Ingegneria, ITALY

## Abstract

Objective characterization of human balance remains a challenge and clinical observation-based balance tests during physical rehabilitation are often affected by subjectivity. On the other hand, computational approaches mostly rely on center of pressure (COP) tracking and inverted pendulum models, which do not capture the multi-joint and muscle contributions to whole-body balance. This study proposes a novel musculoskeletal modeling and control methodology to investigate human balancing capabilities in the center of mass (COM) state space. A musculoskeletal model is integrated with a balance controller trained through reinforcement learning (RL) to explore the limits of dynamic balance during postural sway. The RL framework consists of two interlinked neural networks (balance recovery and muscle coordination) and is trained using Proximal Policy Optimization (PPO) under multiple training strategies. By exploring recovery from random initial COM states with a trained controller, a balance region (BR) is obtained that encloses successful state-space trajectories. Comparing BRs obtained from different trained controllers with the analytical postural stability limits of a linear inverted pendulum model, we observe a similar trend in COM balanced states, but reduced recoverable areas. Furthermore, the effects of muscle weakness and neural excitation delay on the BRs are investigated, revealing reduced balancing capability in the COM state space. The novel approach of determining regions of stability through learning muscular balance controllers provides a promising avenue for personalized balance assessments and objective quantification of balance capability in humans with different health conditions.

## Introduction

Falls and subsequent injuries pose a significant health risk for the elderly and mobility-impaired populations. Poor balancing capabilities are the leading cause of falls in the elderly population, reducing the overall quality of life of aging patients [1]. Effective balance assessment and rehabilitation are critical components not only to health monitoring and injury prevention in mobility-impaired individuals, but also to the diagnoses of other serious underlying medical conditions. It is difficult to pinpoint a single origin causing balance deficiencies in patients and to assess postural stability through simple isolated measures, since balance is maintained through a complicated network of physiological systems in the body. In most clinical environments, balance assessment is performed as a battery of balance exercises designed to evaluate the patient’s ability to perform selected tasks [2]. However, these balance assessment techniques can be influenced by the subjectivity that is inherent within their observation-based scores. Since accurately assessing balance and recovery is a multi-faceted problem, introducing objective and customized approaches into treatment plans can aid in providing better care.

To create more objective assessment criteria, instrumented platforms are often used to obtain kinematic or dynamic measures of a subject’s posture, such as the Center of Pressure (COP) sway and sway velocity [3] or Center of Mass (COM) sway [4]. However, these measures alone are not direct and comprehensive indicators of the balance ability of a subject, since they do not capture the multi-joint and muscle contributions to the whole-body dynamics, necessary to evaluate the specific limits of dynamic balance of the system. Approaches involving the partitioning of a state space of a legged system [5] into viable and failed regions have also been explored, among which the COM state space has shown to be successful in characterizing the limits of dynamic balance (i.e., balance stability) of legged systems [6]. Based on the state space and general viability kernel concepts [7], a constrained optimization problem was developed for the biomechanical analysis of balance using the COM state (position and velocity) [6], ultimately partitioning the state space into two sets: balanced and unbalanced states. A balanced state is defined as a viable COM initial condition, such that at least one whole-body controlled trajectory exists that brings the system from the balanced state to the upright static equilibrium, without necessarily altering its foot stance (e.g., stepping, falling) [8]. Through this, a balance region (BR) can be constructed to describe a collection of COM states that serve as the necessary condition for the given system to be balanced [6]. This region-based analysis provides a more comprehensive understanding of balance, when compared with the traditional COP- and COM-based metrics, by providing an estimate of the subject-specific limits of balance that consider relevant physical constraints through complex multibody dynamics and kinematics. Additionally, these regions can be employed as reference thresholds to formulate metrics, such as boundary and state margins, that provide a quantification of a subject’s balancing ability [9, 10]. Intermittent control has been presented as an approach representative of the non-continuous nature of balance control in the phase plane, emphasizing the active control of the ankle joint based on the position of the state within the phase plane at any given time [11]. This intermittent control strategy has been demonstrated as a result of reinforcement learning [12] and has been shown to align with experimental outcomes of balance [11], offering insight into the switching nature of balance control.

The above gross motion-based analyses and multi-body dynamics approaches can be useful in evaluating the necessary condition for balance stability and measuring postural outcomes. However, they lack the capability to examine the underlying muscular control mechanism that is physiological and has a profound influence on balance in humans. The incorporation of muscle models can help account for physiological effects at the muscle level [13], though recent efforts are being made to investigate the neural component as well [14]. Musculoskeletal (MSK) models help bridge the gap between multibody dynamics approaches and physiology by linking joint actuation with individual muscle actions. Nevertheless, they have not yet been considered in the computational multibody dynamics approaches for the objective characterization of human balance. For instance, disorders at the neuromusculoskeletal level (e.g., Parkinson’s Disease) can affect individual muscle parameters, such as muscle force generation and muscle activation, leading to a change in balance response [15, 16]. Therefore, for a more in-depth and physiologically relevant understanding of balance stability, the use of an MSK model is of particular importance.

Developing a real-time muscle-based balance controller is an effective approach to understand how humans maintain balance and interact with their environment (e.g., in response to posture or force perturbation). Balance control with muscles is much more complicated than traditional torque-based control due to the redundancy in muscles and intricate physiological response of muscle neural commands. Traditional MSK control problems rely heavily on experimental data, such as muscle electromyography (EMG) or motion capture [17, 18], mainly due to the redundancy in muscle control. Other approaches also utilize traditional Proportional-Derivative (PD) control to develop neural controllers for MSK models [19], but these are limited in their simplification of muscle dynamics [20]. Additionally, although reflex-based muscle control has been used in balance studies under perturbations [19,21], these methods typically require complex optimization of PD gains across all muscles and have not been applied for comprehensive stability region analysis. Recently, researchers have been using deep reinforcement learning (RL) and neural networks to control neuromusculoskeletal models to learn complex human movement skills [22–26]. Unlike traditional bipedal control [7,27] and PD control of MSK models, RL-based controllers can account for many factors that comprise balance and its assessment. Combining an MSK model with an RL framework can predict balance capabilities while including characteristics (e.g., muscle strength, neural excitation-activation delay) of specific individuals or patients. Additionally, to improve the performance and real-world applicability of RL agents, considering a baseline model based on offline optimization (e.g., through model predictive control) to initialize the RL model and allowing it to continue learning online could help minimize the time needed for the RL agent to become viable [28, 29].

In this study, we employ an MSK model controlled by a balance controller trained through RL to study human balance. Such a robust muscle controller enables a forward dynamics approach that brings the human from a perturbed state to a balanced upright posture and establishes the BR through a thorough exploration of the COM state space. By studying the BR attainable by RL-trained muscle controllers, we can gain deeper insights on neuromuscular control of balance and the confounding factors, such as muscle weakness and neural delay, which contribute to the deterioration of balance in humans. This work aims to provide a novel and customized approach towards constructing controller-based BRs for the characterization and assessment of balance in humans.

## Materials and methods

A muscular controller for postural balance recovery is developed using RL and is implemented with an MSK model to assess stability in the COM state space. The RL training environment adapts the structure of two neural networks proposed in the MASS framework developed by Lee at al. [25], originally designed to track activities from motion capture data. Our approach instead utilizes a desired equilibrium state (static, upright posture in double stance) as the target during training, thus eliminating the need for tracking experimental data. Physics- and balance-inspired rewards are also formulated for the RL algorithm to effectively guide the learning process. After training, the controller is tested iteratively at varying initial states to generate a balance stability region in the COM state space. The presented RL-based controller can control individual muscles and instantaneously respond to the state of the human with the intent to bring it to equilibrium.

### Musculoskeletal model and whole-body dynamics

**Fig 1:**
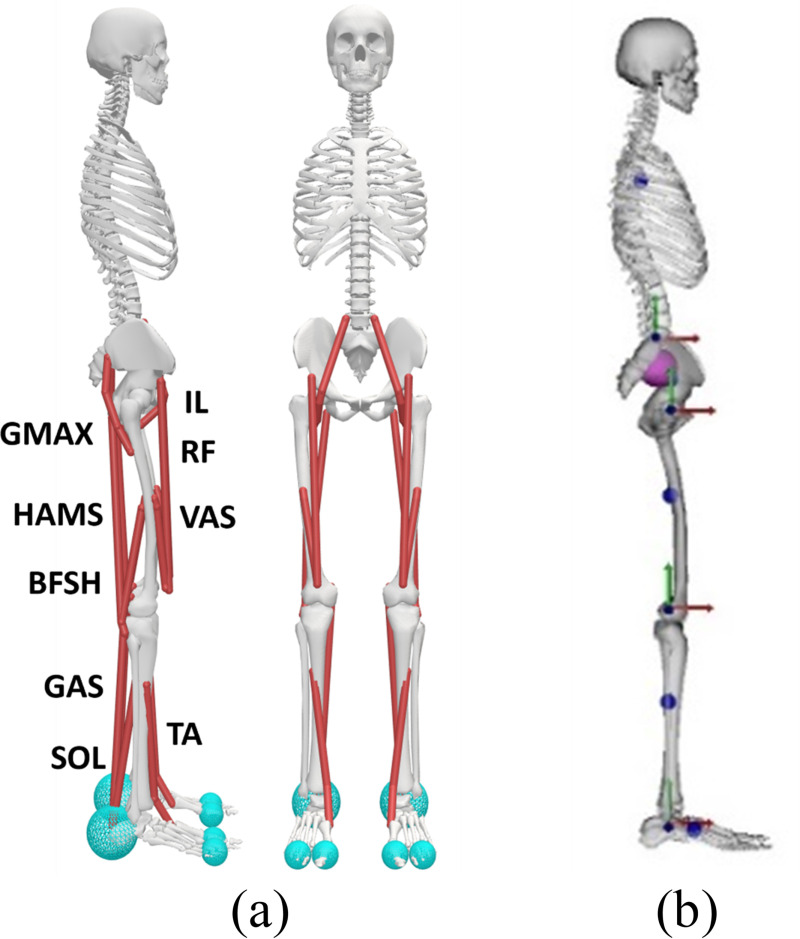
(a) Musculoskeletal model with 18 muscles: gluteus maximus (GMAX), iliopsoas (IL), hamstrings (HAMS), rectus femoris (RF), vasti (VAS), biceps femoris short head (BFSH), gastrocnemius (GAS), soleus (SOL), and tibialis anterior (TA). (b) Joint axes for ankle, knee, hip, and lumbar are presented in red (x), green (y), and blue (z) axes with the z-axis as the rotational axis for all joints. COM locations of individual bodies (foot, shank, thigh, pelvis, torso) are presented as blue spheres on the model. The whole-body COM (pink) is also presented and largely obscures the pelvis COM due to their near coincidence.

A 3D MSK model (Fig 1) with 9 bilateral muscles (totaling 18 muscles) is adapted from the gait10dof18musc model available in the OpenSim repository [30], where the pelvis joint is constrained to a planar motion to simulate whole-body balance in the sagittal plane. The model possesses 10 degrees of freedom (3-DOF pelvis joint, 1-DOF lumbar joint, and left/right symmetric 1-DOF hip, knee, and ankle joints). In this work, the lumbar joint is locked to focus the analysis on balancing strategies enabled by lower-limb joints and corresponding muscles. The simulation environment uses DART (Dynamic Animation and Robotics Toolkit), an open-source physics engine used mostly for robotics and computer graphics [31]. DART was chosen due to its efficiency and accuracy with a reduced coordinate approach and has been extended in this study to load MSK models and compute muscle mechanics.

For each foot, three contact spheres are positioned to establish contact with the ground. Specifically, one sphere is located at the heel (4.9 cm behind the ankle joint along the anteroposterior (AP) direction), while the other two spheres are positioned near the toe joint (15 cm in front of the ankle joint in the AP direction); the foot COM is located 5.1 cm in front of the ankle joint. When the model is in the upright standing position, the height of the COM is measured to be 98.8 cm. In the original gait10dof18musc model, a Millard muscle model with elastic tendon was employed [32]. However, for computational efficiency, we opted for a rigid-tendon muscle model, which not only significantly speeds up computations but also often yields comparable accuracy [32]. Our muscle model closely resembles the one utilized in MuJoCo [33], augmented with the incorporation of the pennation angle.

The muscle model takes the muscle force-length-velocity relations and fiber pennation angle into consideration when determining muscle force [32,34], which is shown as follows:


$$F_{M}=F_{max}\cdot \left[a\cdot F_{L}\left(l\right)\cdot F_{V}\left(\dot{l}\right)+F_{p}\left(l\right)\right]\cdot \cos\left(\alpha \right)$$ (1)

where $F_{max}$ is the muscle-specific maximum isometric muscle fiber force, *a* is the muscle activation ranging from 0 to 1, *α* is the fiber pennation angle, and *l* is the normalized muscle length with respect to the optimal fiber length. Additionally, $F_{L}\left(l\right)$ and $F_{V}\left(\dot{l}\right)$ are the normalized force-length and force-velocity curves, respectively. $F_{p}\left(l\right)$ represents the normalized passive force-length relationship. The muscle activation (*a*) is governed by a first order excitation-activation dynamics equation:


$$\dot{a}=\frac{u-a}{\tau \left(u,a\right)}$$ (2)

where *u* is the muscle excitation (motor command) and *τ* is the delay time, which is computed as [32]:


$$\tau \left(u,a\right)= \{\begin{array}{cc} \tau_{act}\left(0.5+1.5a\right) & u-a>0\\ \tau_{deact}/\left(0.5+1.5a\right) & u-a\leq 0 \end{array}$$ (3)

where $\tau_{act}$ and $\tau_{deact}$ are muscle activation and de-activation time constants with a default of 0.01 and 0.04 seconds, respectively. The delay between muscle excitation and muscle activation can be interpreted as the time required for the excitation signal to propagate from the motor neurons to the muscle fibers and for the subsequent physiological processes to occur, resulting in muscle contraction. This dynamics equation is solved through integration, while both excitation and activation are clamped within [0,1].

The human musculoskeletal dynamics is mapped in the joint space, where it is governed by the Euler-Lagrangian equations using generalized coordinates:


$$M\left(q\right)\overset{"}{q}+c\left(q,\dot{q}\right)=J_{M}^{T}F_{M}+J_{ext}^{T}F_{ext}$$ (4)

where *q*, $\dot{q}$, $\overset{"}{q}$ are the vectors of joint angles, angular velocity, and angular accelerations, respectively. $F_{ext}$ is the $R^{3}$ vector of external forces (such as contact forces) and $F_{M}$ is the $R^{n}$ vector of muscle forces (*n* is the number of muscles) that depends on the muscle activation vector $a=\left(a_{1},a_{1},\cdots a_{n}\right)$. $M\left(q\right)$ is the generalized mass matrix, and $c\left(q,\dot{q}\right)$ is the generalized bias force accounting for the Coriolis and gravitational forces. $J_{M}$ and $J_{ext}$ are the Jacobian matrices which map the muscle and external forces into generalized joint torques. Since the muscle force is linear with respect to the activation as shown in Eq. 1, the muscle forces are computed as:


$$F_{M}=\frac{\partial F_{M}}{\partial a}a+F_{M}\left(0\right)$$ (5)

The corresponding generalized joint torques from muscles are defined by:


$$J_{M}^{T}F_{M}=J_{M}^{T}\left(\frac{\partial F_{M}}{\partial a}a+F_{M}\left(0\right)\right)$$ (6)

When implemented in the RL framework, the dynamics of the MSK model are integrated using a forward dynamics approach with muscle excitations provided by the muscle coordination neural network. During the forward simulations, kinematic constraints such as joint limits are enforced and contact forces within the friction cone are solved with a linear complementarity problem (LCP) formulation [31].

For comparative analysis, here we briefly introduce the extrapolated COM (XcoM) that is often used to establish the balance stability region of a Linear Inverted Pendulum (LIP) model [35]:


$$XcoM=x+\frac{v}{\omega }$$ (7)

where *x* is the horizontal COM position in the sagittal plane, $v=\dot{x}$ is the horizontal COM velocity, and $\omega =\sqrt{g/L}$ is the natural frequency of the LIP, where *g* is the gravity constant and *L* is the height of the LIP. For the human MSK model, *L* is the whole-body COM height in upright standing (98.8 cm). The stability condition for maintaining balance in the LIP framework necessitates that the XcoM remains enclosed within the base of support (BoS) of the legged system [35]. To define the BoS, we utilize the maximum range of COP displacement between the heel and toe contact spheres. By equating the expression of the XcoM (as per Eq. 7) to the anterior and posterior edges of the BoS, we establish two parallel lines with a slope of -ω, delineating the dynamic balance limits of the LIP model within the COM state space [10]. This balance within the LIP model is based on several assumptions: a point mass model with a constant COM height, a rigid flat foot, unrestricted friction force, unconstrained ankle motion, and ankle torque indirectly regulated by constraining the COP within the BoS.

### Reinforcement learning framework

**Fig 2:**
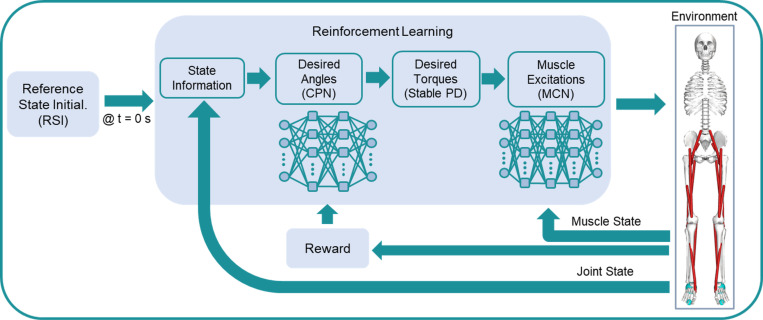
Overall RL control framework. A random initial state is fed into the algorithm at the start of each episode and two neural networks (CPN and MCN) are used to control the MSK model. RL rewards are computed to update the CPN, and a supervised loss function is used to update the MCN. The random initial state is given via selected ankle position and velocity determined by the Reference State Initialization (RSI) algorithm.

The goal of the RL framework is to train the MSK model to recover balance from random initial states in the COM space, which are indirectly imposed by the angular position and velocity of the ankle joint. This generates a controller that takes as input the human body state information, predicts desired joint angles, and transforms them into desired joint torques through PD control, and outputs muscle excitations as the control command for the physical MSK simulation environment (Fig 2). After the training phase, the controller is tested for balance recovery by using many random initial states, which are classified as either successful or unsuccessful based on whether the model falls or moves its feet, and the successful states are used to build the system’s BR.

The learning environment is the dynamic simulator, where the MSK model interacts with the ground. Control of the environment is achieved through a combination of two multilayer perceptron (MLP) neural networks (Fig 2): one is the muscle coordination network (MCN, $\pi_{\psi }$) and the other is the control policy network (CPN, $\pi_{\theta }$). The agent (CPN) takes as input the human body state information (the aggregations of the 3D positions and linear velocities of the COMs of all bones), then outputs the desired joint angles as the action. The desired joint angles are subsequently transformed to desired joint torques ($\tau_{d}$) through stable PD control [36]. The PD gains, $k_{p}$ and $k_{v}$, are set to 300 and 24.5, respectively [26]. Separating joint motion control and muscle excitations into two networks allows these networks to learn and operate at different frame rates. The joint network learns at a low frame rate (e.g., 30 Hz), while the muscle network learns and operates at the rate of forward dynamics simulation (e.g., 600 Hz), following the approach proposed by Lee et al. [25]. In addition, the joint network control policy $\pi_{\theta }(a|s)$ is a stochastic policy, whereas the muscle network is a deterministic policy. These networks interact with each other to achieve maximum rewards in RL.

### Muscle Coordination Network (MCN)

The neural network used for learning muscle activations is a deterministic policy $a=\pi_{\psi }\left(\tau_{d},s_{muscle}\right)$, where the network parameters *ψ* are learned through regression by supervised learning. The muscle network is set up as an MLP network with 3 hidden layers (n =  512, 256, 256 nodes) and the loss function for training is:


$$Loss\left(a\left(\psi \right)\right)=E\left[{\left\|\tau_{d}-J_{M}^{T}\left(\frac{\partial F_{M}}{\partial a}a\left(\psi \right)+F_{M}\left(0\right)\right)\right\|}^{2}+w_{reg}{\left\|a\left(\psi \right)\right\|}^{2}\right]$$ (8)

The first term is used to minimize the discrepancy between the desired torques and the muscle-produced joint torques under the predicted activation $a\left(\psi \right)$. The second term is a regularization for large muscle activations. To enforce normalized muscle activations within $\left[0,1\right]$, a bounded activation function is used for the output (i.e., Tanh function followed by a ReLU function). The MCN predicted $a\left(\psi \right)$ is fed to the simulation environment as the muscle excitation (*u*), since the activation cannot be directly set due to the excitation-activation delay constraint (Eq. 2).

### Control Policy Network (CPN)

The CPN acts as the main RL agent controlling the MSK model’s actions, based on its accumulated rewards. As the RL agent interacts with its environment, its actions are scored using a reward and the agent is updated based on the action’s reward. At each time step *t*, the agent’s state $s_{t}$ is observed and an action $a_{t}$ is selected according to its control policy $\pi_{\theta }(a_{t}|s_{t})$ with *θ* being the weights and bias of the neural network. The control policy is learned by maximizing the discounted sum of reward ($r_{t}$):


$$\pi^{⋆}=\arg\underset{\pi }{\max}E_{\tau \sim p(\tau |\pi )}\left[\overset{T-1}{\underset{t=0}{\sum }}\gamma^{t}r_{t}\right]$$ (9)

where $\gamma \in \left(0,1\right)$ is the discount factor, *τ* is the trajectory over time $\left\{\left(s_{0},a_{0},r_{0}\right),\left(s_{1},a_{1},r_{1}\right),...\right\}$, p(τ|π) is the likelihood of that trajectory *τ* under the control policy *π*, and *T* is the horizon of an episode.

The RL framework is trained with the Proximal Policy Optimization (PPO) algorithm [37], which is a model-free policy gradient algorithm widely used for continuous control problems. PPO updates the control policy’s parameters (*θ*) using the expected return’s gradient with respect to the parameters. The agent learns to increase its reward by modifying the parameters *θ* of the network. The CPN is implemented as an MLP network with 2 hidden layers of 256 nodes each (Fig 2). A desired equilibrium state is provided as the target posture for learning balance recovery, which is set as the whole-body COM situated right on top of the foot COM vertically (hip and knee =  0°, ankle =  5.58°). In addition, the present network does not require a phase variable (ratio between current simulation time and end time of reference motion [25]) as an input.

### Reward functions

At any time instant *t*, the reward function $r_{t}$ for the RL algorithm is designed to drive the MSK model to reach the target state by including a target posture reward $r_{t}^{p}$, a torque reward $r_{t}^{torque}$, a body upright reward $r_{t}^{up}$, and an LIP-inspired reward based on the XcoM, $r_{t}^{xcom}$, and is defined as:


$$r_{t}=w^{p}r_{t}^{p}+w^{torque}r_{t}^{torque}+w^{up}r_{t}^{up}+w^{xcom}r_{t}^{xcom}$$ (10)

where $w^{p}=1.0$, $w^{torque}=0.1$, $w^{upt}=0.1$, and $w^{xcom}=0.1$ are their respective weights. The target posture reward is designed to match the target posture with the actual joint angles:


$$r_{t}^{p}=\text{exp}\left(-\sigma_{p}\underset{j}{\sum }{\left\|{\hat{q}}_{t}^{j}-q_{t}^{j}\right\|}^{2}\right)$$ (11)

where *j* is the joint DOF index, ${\hat{q}}_{t}^{j}$ is the desired joint variable for the target standing posture, and $\sigma_{p}=2.0$. The torque reward is included to help reduce energy consumption of the joints:


$$r_{t}^{torque}=\text{exp}\left(-\sigma_{torque}\underset{j}{\sum }{\left\|\tau_{t}^{j}\right\|}^{2}\right)$$ (12)

where $\sigma_{torque}=0.001$. The upright posture reward is set to be:


$$r_{t}^{up}=\text{exp}\left(-\sigma_{up}{\left\|p_{head}^{x}-p_{pelvis}^{x}\right\|}^{2}\right)$$ (13)

where $\sigma_{up}=100$ and $p_{pelvis}^{x}$ is the *x* (horizontal) position of the pelvis, $p_{head}^{x}$ is the *x* position of a point on the head which equals to $p_{pelvis}^{x}$ when pelvis tilt angle is zero (i.e., torso is upright). The XcoM reward is provided by:


$$r_{t}^{xcom}=\text{exp}\left(-\sigma_{xcom}{\left\|XcoM_{t}-XcoM_{target}\right\|}^{2}\right)$$ (14)

where $\sigma_{xcom}=40$ and $XcoM_{target}$ is set as the *x* position of the foot’s COM. The chosen target for XcoM effectively encourages the muscle controller to maintain the XcoM within the BoS and toward the center of the foot for a larger margin of stability during balance recovery. Particularly, during static posture, the XcoM position is also equivalent to the COP and setting it to the foot COM presumably ensures a stable posture.

### Training strategy

In the work by Peng et al. [38], two specific components, reference state initialization (RSI) and early termination, are identified to be critical for achieving highly dynamic motions from mimicking a reference motion. In this study, we employ a novel RSI even in the absence of a reference motion. By leveraging RSI, the agent can benefit from a diverse and informative distribution of states, which can effectively guide its training process. The integration of RSI during training encourages exploration of the COM state space by exposing the controller to different initial states, which may range from relatively easy to highly challenging, or even infeasible, conditions across different episodes. Initial joint states for each training episode are randomly selected from normal distributions for both angular position (mean: $\mu_{p},$ standard deviation (SD): $\sigma_{p}$) and velocity (mean: $\mu_{v},$ SD: $\sigma_{v}$) of the ankle joint to encourage exploration of the COM state space. These distributions are informed by the balance dynamics of the LIP model, which provides a theoretical reference of what states a standing posture can potentially be recovered from (under the LIP balance region assumptions) [35,39] and consequently maximizes the potential for success in training.

The other joint DOFs (hip and knee) are set to zero for the initial posture, so that the model has straight legs in the beginning; during simulation, all lower-body joints are allowed to move. For a given pair of ankle angle and angular velocity, we use an optimization-based inverse kinematics method to find pelvis position and tilt angle, and their velocities, such that the feet remain stationary (zero velocity) at the same locations. The current initial conditions on ankle angles and velocities likely induce ankle strategies for balance recovery at the beginning; however, during the forward dynamics simulations, all joints are free to move. Consequently, alternative strategies may emerge during the later phase of balance recovery.

An early termination strategy offers an alternative means of shaping the reward function in order to discourage undesirable behaviors and can function as a curating mechanism that favors data samples that may be more pertinent to a given task. Here, the following events are used as early termination conditions:

- Fall: pelvis height is lower than a threshold, which is set to 0.8 m, corresponding to an ankle angle of 40°.
- Foot slide: any foot contact moves more than 1 cm in the AP direction.
- Foot lift: any foot contact lifts more than 1 cm in the vertical direction.

During training, each episode is a musculoskeletal simulation that ends at 10 seconds unless it is terminated earlier. The hyperparameters used in this study are taken from [26].

To encourage convergence for a robust and natural human balance controller, three controllers are trained using different approaches: 1) RSI with random initial ankle angle ($\sigma_{p}\neq 0$) and zero initial ankle velocity ($\mu_{v}=\sigma_{v}=0$), 2) RSI with random initial ankle angle and velocity $\left(\sigma_{p}\neq 0,\sigma_{v}\neq 0\right)$, and 3) a two-step curriculum learning (CL) process by first training with zero starting velocity ($\mu_{v}=\sigma_{v}=0$) and then continuing with non-zero random velocity $\left(\sigma_{p}\neq 0,\sigma_{v}\neq 0\right)$. CL [40] has been used in literature to learn complex human and robot movement skills [22, 23]. In the CL process, we start by learning to balance from an inclined state with zero initial velocity (by setting the initial velocity mean and SD $\mu_{v}=\sigma_{v}=0$). After completion of the first step’s training, the best performing neural networks (with the highest reward or minimal loss) are used as the starting point of the next step, which uses non-zero velocity mean and SD ($\mu_{v}=s\times \theta_{ankle}$, $\sigma_{v}=0.1rad/s$). *s* is a slope factor that selects the velocity mean based on the current sampled initial ankle angle $\theta_{ankle}$ and the slope can be adjusted to follow the slope of the LIP balance region limits. All training is performed on a Linux machine with Intel Xeon CPUs (2.30GHz) and a 16GB Nvidia Quadro RTX 5000 GPU, and each training uses 50,000 iterations and it typically takes around 40 hours to complete.

### Testing and balance region generation

For each training method, the learned balance controller is tested on the MSK model to examine its ability to regain balance from various initial states. During testing, the controller strives to drive the system from a given random initial state to an upright balanced state without triggering the same terminating conditions that were used during training. To generate the BR with the trained controller, 10,000 simulations are performed with an RSI strategy equal to that of training method 2. The number of performed simulations is selected to ensure densely sampled initial states within reasonable computational time. The outcome of each simulation is recorded as “successful” if it runs to the end of the specified episode time (10 seconds) without triggering any termination condition; otherwise, it is recorded as “unsuccessful”. For every successful simulation, the corresponding initial COM position and velocity are stored as a point of the system’s BR. A point-based BR (PBR) is generated from the envelope of all successful initial COM states. However, during the course of dynamic balance recovery, a COM state trajectory may pass through states outside of the PBR, since the PBR contains only initial successful states with straight legs (imposed by RSI); all points on a successful trajectory (COM states at discrete times) lead to balance recovery at the end. Therefore, these points along the successful trajectories should be considered as a part of the final BR. The PBR and (final) BR envelopes are generated from a point cloud using the alpha shape toolbox (https://github.com/bellockk/alphashape) in Python, which finds the bounding polygon of a point cloud.

## Results

The neural networks were trained using the three different methods outlined in the training strategy (1: RSI with random initial ankle angle and zero velocity; 2: RSI with random initial ankle angle and velocity; and 3: two-step CL). For the first method, the following parameters were used: $\mu_{p}=0.09745 rad$ corresponding to the target ankle angle ($\text{5}{\text{.58}}^{\text{°}}\text{, plantarflexion)},$$\sigma_{p}=0.1 rad,s=\text{0},\mu_{v}=\text{0},\text{and}\sigma_{v}=\text{0}$. For the second method, the same $\mu_{p}$ and $\sigma_{p}$ were used, while *s* was set to ω=3.15 s^-1^ and $\sigma_{v}$ was set to 0.1 rad/s. As for the third (two-step CL) method, the training with method 1 was repurposed as the first step and the second step involved using the best outcomes from the first step, then training was performed again using identical parameters from method 2.

**Fig 3:**
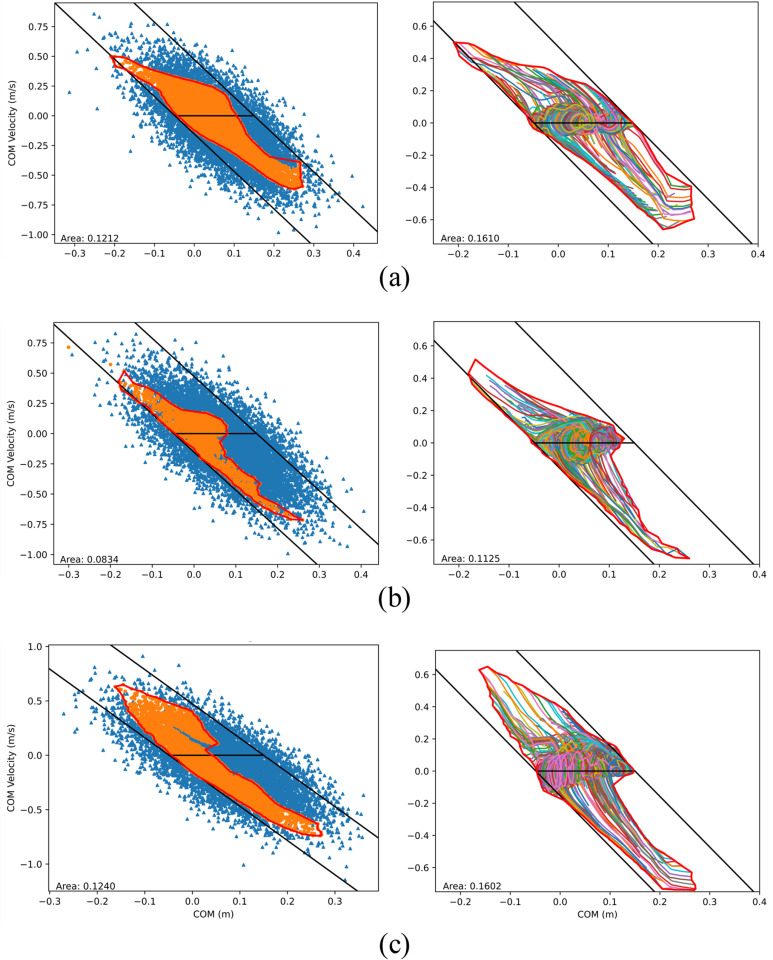
COM state space balance regions (enclosed by the thick red curves) based on the testing of the learned controllers trained with (a) method 1 (zero starting velocity); (b) method 2 (non-zero velocity); and (c) method 3 (CL). Left: Initial COM states (points) of successful trials (orange markers) and unsuccessful trails (blue markers) and generated PBR envelopes from the successful trials. Right: COM state trajectories of selected successful trials and generated final BRs.

Using the learned controllers from these three methods, we conducted tests to generate their BRs; for each controller, 10,000 dynamic simulations were run with initial states randomly sampled from the same normal distributions $(\mu_{p}=0.09745,\sigma_{p}=0.1,s=-3.15,\mu_{v}=\text{0},\text{and}\sigma_{v}=0.1)$ as those used for the second training strategy. The overall success rate of each controller is calculated as the number of successful simulation trials with respect to the total number of trials conducted, which resulted in 59.59%, 16.31%, and 41.60% for the three training methods, respectively. Successful (orange markers) and failed (blue markers) simulation trials of the three controllers are illustrated in Fig 3. Within the PBR envelopes (red curves), scattered failed points exist—most of which are not visible due to rendering. Method 1’s PBR envelope contains 5959 successful and 74 failed points, the latter being mostly located near the bottom right corner where the PBR is concave. This indicates an “internal” success rate within the PBR equal to 5959/(5959 + 74) =  98.77%; smaller success rates within the PBRs for methods 2 and 3 are found (42.73% and 87.38%, respectively). The area of each method’s PBR is also listed in the figure; methods 1 and 3 have comparable areas that are both over 45% larger than the area of method 2.

No PBR includes the entire set of static equilibrium points (i.e., points on the horizontal black line in Fig 3, containing the states with zero COM velocity and COM position within the contact limit [10]), for which the COM ground projection coincides with the COP. The placement of the contact spheres in the current contact model limits the COP position within the functional BoS, which has an AP range of [-4.9, 15] cm (heel to toe joint). This is slightly smaller than typical foot size, excluding the possibility of the system to maintain a static posture with a COM projection beyond the toe joint. However, the set of static equilibrium points in the PBRs is even smaller than this range (19.9 cm), particularly in the anterior direction. This is possibly due to a combination of multiple factors, such as muscle-actuated joint torque limits, a simplified contact model, and the chosen initialization strategy for muscle activation at the beginning of each episode during training. Among the three methods, method 1 covers a much wider range of static equilibrium points.

All successful trajectories lead to balance recovery, and all points along such trajectories should be included in the system’s final BR. The final BRs were constructed from a selection of successful COM trajectories in each of the three cases (Fig 3). For computational efficiency, only trajectories originating from the boundary points of the PBR and from an additional 100 randomly chosen successful internal points are included in the generation of the final BR. The alpha shape envelopes for all cases are generated using a shape parameter of 15.

A trend like the PBRs is observed in the areas covered by the final BR: method 2 covers the smallest area, whereas method 1 has a comparable area to that of method 3. Contrary to the PBRs, the BRs in all three methods mostly cover the entire set of static equilibrium points, indicating that for any COM ground projection within the base of support there exists at least one balanced trajectory. The states included in the BR belong to highly dynamic balancing trajectories, taking advantage of the presence of inertial forces [10] and overcoming the previously mentioned factors limiting the PBRs.

The bounds of the LIP stability region were determined using the locations of the toe and heel contact spheres to define the LIP effective BoS, i.e., $u_{max}=\text{15 cm}$ and $u_{min}=-4.9\text{cm}$. The LIP stability limits, subject to their aforementioned assumptions, are compared with the BRs (Fig 3). The zero COM *x*-position coincides with the projection of the ankle joint. All BRs largely fall within the analytical bounds, exceeding them only in few instances; the model in this study is multi-segmental, as opposed to LIP, and can likely exhibit some angular momentum strategy for balance recovery [10].

### Balance recovery from forward and backward lean

**Fig 4:**
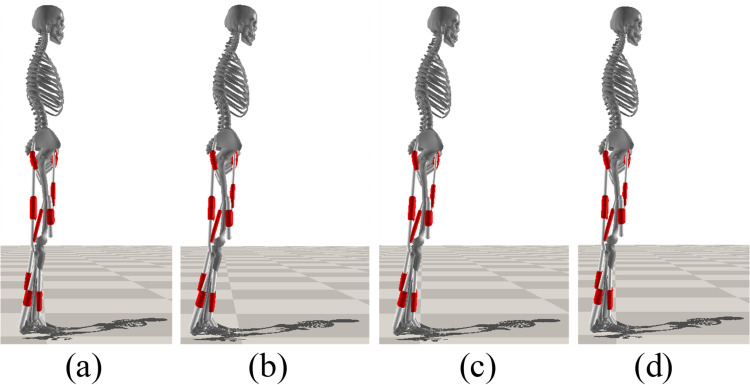
Mean postures at 10s for (a) method 1, COM position: 0.049 ±  0.023 m; (b) method 2, COM position: 0.108 ±  0.033 m; and (c) method 3, COM position: 0.104 ±  0.023 m; and (d) target posture, COM position: 0.051 m. In the figure, muscle tendons are displayed as white cylinders.

To investigate the outcome of the trained controllers on joint kinematics, we analyzed the joint angles of the 100 randomly selected successful trajectories for each of the three cases, as well as the mean final posture at the end of the 10-second simulations. When comparing the mean final postures with the target posture, the outcomes of methods 1, 2 and 3 showed deviations in the COM *x*-position of -0.2 cm, 5.7 cm, and 5.3 cm, respectively. The mean final postures of the MSK model achieved with the controller trained using method 1 resulted in a posture closest to the target posture (Fig 4). It is evident that the final posture resulting from method 1 is more aligned with a natural upright stance, when considering the final COM and joint states, whereas the final postures obtained from methods 2 and 3 exhibit increased forward inclination compared to the target posture. Considering the success rate of PBRs (overall and internal), shape and coverage of BRs, and mean final posture, the controller trained using method 1 (random initial position and zero initial velocity of the ankle joint) appears to be more robust and performs the best.

**Fig 5:**
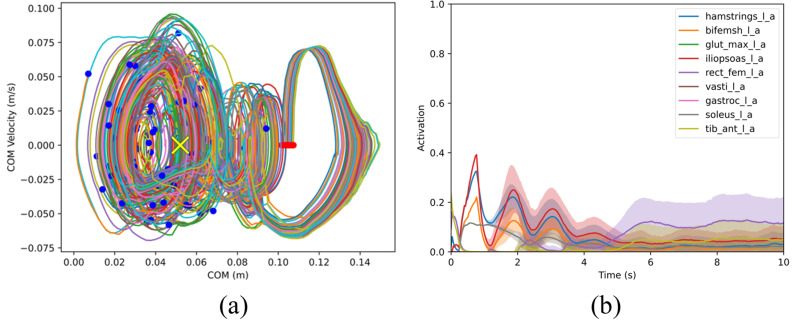
(a) COM trajectories of balance recovery from forward lean using a controller trained with method 1. Red points are the starting positions, blue points are the end states at 10 s, and the yellow cross is the target COM state. For reference, the origin of the COM position is at the same horizontal (x) position as the ankle joint and the x-position of the toe contact point is 0.15 m. (b) Mean and SD plots of muscle activations from forward lean recovery. “_l_a” in the legend text indicates muscle activation on the left side.

**Fig 6:**
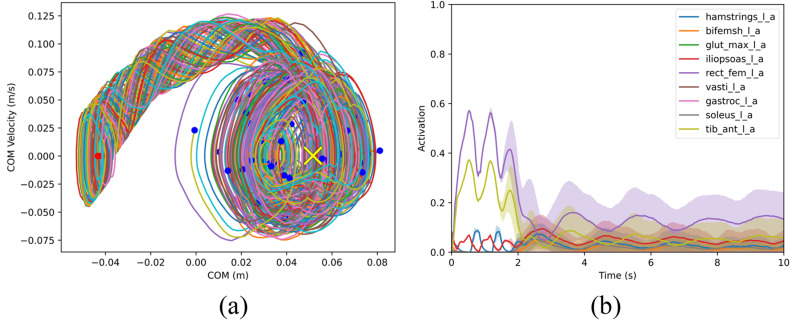
(a) COM trajectories of balance recovery from backward lean using a controller trained with method 1. Red points are the starting positions, and blue points are the end positions at 10 s, where the model is statically stable and within the foot limits; the yellow cross is the target COM state. (b) Mean and SD plots of muscle activations from backward lean recovery.

Using method 1’s trained controller, voluntary balance recovery from forward and backward leans was analyzed. The RSI algorithm was used to obtain 100 random initial states; a mean ankle angle $\mu_{p}=8^{\text{°}}$ (near model’s PBR recovery limit) with ${\text{σ}}_{\text{p}}\text{= 0}{\text{.1}}^{\text{°}}$ and zero velocity was set for forward lean tests and a mean ankle angle of ${\text{μ}}_{\text{p}}\text{= –1}{\text{.45}}^{\text{°}}$ (near backward extreme) with ${\text{σ}}_{\text{p}}\text{= 0}{\text{.1}}^{\text{°}}$ and zero velocity was set for the backward lean tests. In both tests, muscle-controlled dynamic simulations were run to collect 100 successful trials. COM trajectories of these trials for balance recovery from forward lean are presented in Fig 5, along with the time history of muscle activations. The IL demonstrates the highest activation, followed by the HAMS, BFSH, and SOL, the latter being responsible for the ankle plantarflexion torque required in the early stage of balance recovery from a forward lean posture, as expected from standard inverted pendulum model and experiments [41, 42]. The TA exhibits greater activation at the start but has a relatively low level of activation throughout the remaining duration. Contrarily, the GAS displays minimal activation in the early stage of the balance recovery but intensifies its activation towards the end. The GMAX, VAS, and RF are mostly inactive or have very low activation during forward lean balance recovery. Fig 6 presents results for balance recovery from backward leaning. The RF exhibits the highest level of activation, followed by the TA, HAMS, and then the IL. These muscle activation results show the relevant contribution of ankle dorsiflexion torque (through TA activation) in the early stage of balance recovery from backward lean posture, as expected. For the entire duration, both the IL and HAMS demonstrate low levels of activation. Additionally, there is endpoint variance in both Fig 5 and Fig 6 as the model nears its balanced target state—a phenomenon that was also observed in the experimental results of Patton et al. [39].

### Effects of muscle weakness and neural delay

**Fig 7:**
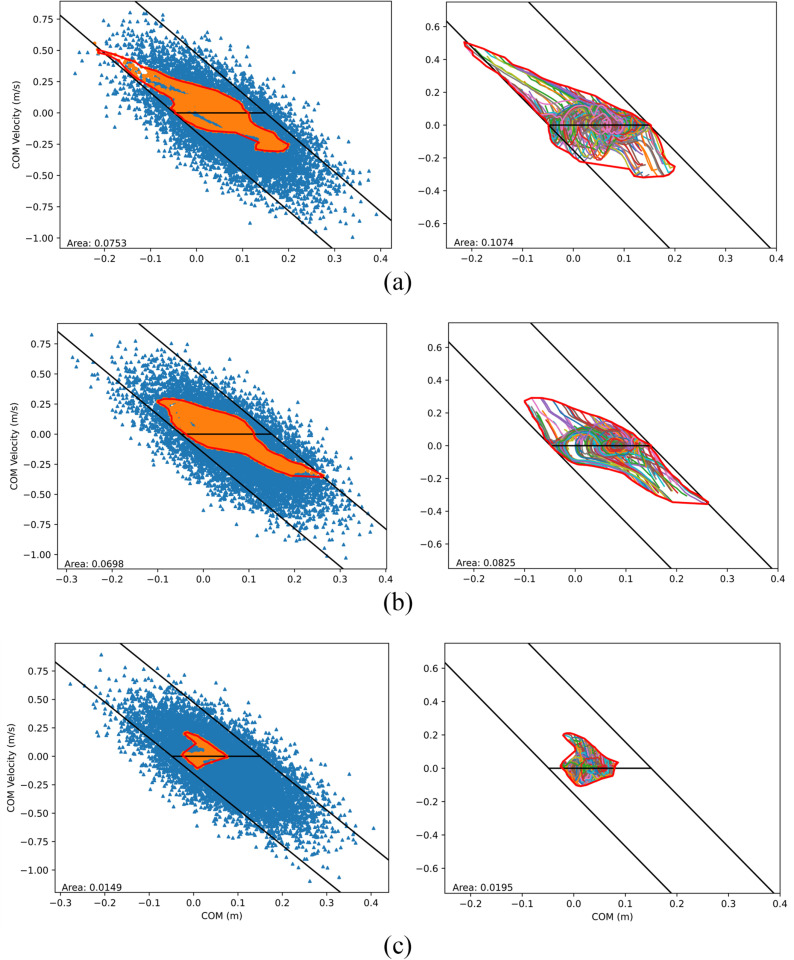
COM state space balance regions for the learned controller trained with method 1 and modified muscle properties. (a) Maximum isometric fiber forces of all muscles were reduced by 30% of their original strength; (b) Maximum isometric fiber forces of muscles on the left side were reduced by 30% of their original strength; (c) Maximum isometric fiber forces of muscles on the left side were reduced to 0% for all muscles.

**Fig 8:**
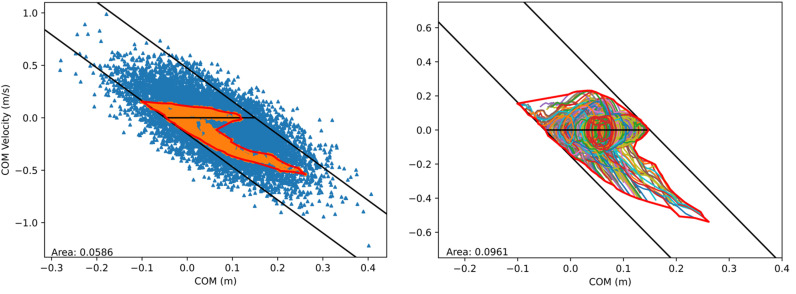
COM state space PBR and BR for the learned controller trained with muscle activation and deactivation time increased by 50%.

Aging and neuromuscular disorders often induce significant changes to muscle physiological properties that affect people’s balance; it has been shown that muscle fiber’s maximum isometric force and contraction velocity decreases while activation and deactivation time constants increases [43, 44]. To investigate the effect of muscle degradation on balance recovery, we conducted tests by modifying muscle properties in several ways. In the first case, maximum isometric fiber forces of all muscles were reduced by 30% to simulate muscle weakness from aging. In the second case, maximum isometric fiber forces of muscles on the left side only were reduced by 30% (hemiparesis). In the third case, maximum isometric fiber forces of muscles on the left side were reduced to 0% of their original strength to simulate complete loss of muscle strength on one side (hemiplegia). In the two latter cases, the maximum isometric fiber forces of muscles on the right side were kept at their original strength; due to the asymmetry, we changed the 3-DOF planar root joint at the pelvis to a 6-DOF free joint, which enables full 3D global translation and rotation. The controller for each case was trained using method 1, then tested with random ankle states. A comparison of both PBRs and BRs for these three cases is presented in Fig 7. To investigate the effect of muscle activation and deactivation time (Eq. 3) on balance recovery, we increased both time durations by 50% to simulate a longer neuromuscular response time (i.e., neural delay). We conducted the same training process to obtain a new controller and tested it to generate new BRs (Fig 8). Compared to the BRs in Fig 3(a), the new BRs are much smaller in covered area, particularly in the region above the zero-velocity line, suggesting that the controller’s performance is compromised when recovering from large posterior sways.

## Discussion

The proposed balance controller trained by the RL framework represents a novel method to explore the limits of dynamic balance of human standing posture, under comprehensive kinematics, contact, and muscle activation constraints. To the best of our knowledge, this work is the first known attempt to generate BRs in the COM state space using a MSK model driven by RL. Our RL approach involved two decoupled yet interconnected neural networks, similar to the method employed by Lee et al. [25]. Unlike the work by Lee et al., our methodology revolves around a predetermined target posture for balance recovery, eliminating the need for motion tracking. Additionally, we use a well-established OpenSim model that has been calibrated to ensure accuracy in muscle paths and parameters. Furthermore, we employ a more sophisticated muscle model than the relatively simple one used in Lee et al., thereby enabling the accurate capture of muscle parameters and mechanics.

In our RL framework, we developed various physics- and balance-inspired rewards for controlling balance recovery: reaching a target balanced posture, maintaining an upright upper body, and utilizing an LIP model-based balance criterion known as XcoM. We conducted an ablation study by removing selected rewards from the total reward in Eq. 9 and trained additional controllers with method 1. Without the XcoM reward, the generated system PBR has an overall success rate of 17.29% and a success rate of 51.75% within the PBR. Without the upright posture reward, the PBR has an overall success rate of 20.46% and a success rate of 56.77% within the BR. Without the target posture reward, the generated PBR has an overall success rate of 26.19% and a success rate of 94.79% within the PBR. Excluding the torque reward led to a substantial decrease in the overall success rate at 2.19% and an internal success rate of 30.12%. The notably low success rate without the torque reward underscores the importance of regulating joint torques, which directly connect both CPN and MCN.

To explore the COM state space during balance recovery, we devised a novel procedure for RSI, which was utilized in both training and testing. We also investigated the use of early termination and CL to enhance the efficiency and convergence of balance recovery controllers during training. We found that RSI and early termination were crucial in achieving robust balance recovery controllers, aligning with the observations made by Peng et al [38]. We explored three distinct training methods, where each utilized different RSI strategies or CL. The three training methods (zero initial velocities, non-zero initial velocities, and curriculum learning) are compared to identify the best approach to employ for all tested conditions (healthy, reduced strength, hemiparesis, hemiplegia).

The controller generated from the first method (random initial ankle angle with zero velocity) exhibited the best overall performance in terms of robustness (higher success rate) and coverage of the recoverable COM state space (i.e., greater BR area), as compared to the other two methods. It might initially appear unexpected that method 1 (trained with zero initial velocity) produces better control results when tested and adeptly handles non-zero velocity initial conditions, outperforming both method 2 and 3. A plausible reason is that with increasingly complex initial conditions (i.e., dynamically stable vs. statically stable [6,45]), the training encounters more failures and does not learn as efficiently as method 1, leading to controllers that lack optimality and robustness. Implementing more refined or new training procedures could potentially improve training outcomes, which for example could be done through progressively increasing the difficulty of the RSI, considering desired initial or final acceleration or COP conditions, and fine-tuning the early termination conditions.

When comparing the successful COM states shown in the PBRs and the corresponding final BRs generated from the trajectories, we can observe that the BRs generally cover more COM state space areas than the PBRs. Note, in the RSI, the initial states are only specified for joint angle and angular velocities; however, initial muscle states (i.e., activations) are also important in determining the balance outcome. One choice is to set muscle activations to zero at the beginning and let them ramp up with the MCN predicted excitation. Since this introduces a delay in muscle response to the initial inclined state, we set the muscle activations to be the same as the excitations to mimic the effect of anticipation. Therefore, two coincident COM states in the PBR and BR could indicate distinct overall system dynamic states that consider muscles and result in different balance outcomes.

Ideally, it is desired to have a universal and highly robust balance controller that can recover from the largest possible area within the COM state. This likely requires further study by employing more advanced training methods, which can effectively explore the control space, avoid convergence to local optima, and utilize sophisticated physical models that account for variations in physics properties and uncertainties in human-environment interaction. In the literature, techniques such as domain randomization [24,26,46,47] have been used to enhance robustness of trained controllers. Domain randomization enables the trained controller to better manage inaccuracies and disturbances by incorporating randomized model parameters during training. This strategy equips the RL agent to adapt to model and dynamics variations (such as mass, inertia properties of segments, and muscle strengths), thereby enhancing the controller’s robustness [26]. To mitigate model mismatch between computational and real-world implementations and outcomes, domain or model randomization can also be used to adjust strength, anthropometric parameters, and scaling of the musculoskeletal model [26]; this technique can help create more robust controllers that are less sensitive to model inaccuracies, making them more adaptable to real-world variability. Additionally, our framework could be extended to subject-specific models by incorporating individual anthropometric data and musculoskeletal characteristics (e.g., muscle strength, neural delays); this would help reduce model mismatch and could significantly improve the applicability of our method to real-world diagnosis.

The BRs obtained using various trained RL controllers and the MSK model were largely contained within and aligned with the analytical LIP model-based limits but did not have large COM excursions that deviate too far from the balanced upright posture. This is because the MSK model includes limits of human strength, contact model, and joint range of motions that are not considered in the theoretical model, which, as a result, overestimate the true human capabilities of standing balance, especially for large COM sway. In the ideal LIP model the total area of the analytical limits is infinite in theory, but these limits can be constrained using the friction cone to obtain a BR with appropriate limits [10]. On the other hand, in some instances (near the upright posture) the LIP point mass model may underestimate the subject balance capabilities, which include multi-segmental strategies.

Comparable human COM data for balance recovery is limited in the literature. One notable study by Patton et al. [39] investigated double stance balance recovery of subjects using a force measurement handle to exert anterior momentum. However, their study only recorded the posterior half of the balance recovery trajectories. Compared to their data, our simulated recovery trajectories in the state space (Fig 3, right) reveal pattern similarities. For instance, the trajectories initially travel parallel to the LIP stability boundaries before converging towards the center of the foot. Interestingly, some of our predicted balance trajectories breach the LIP boundary lines (Fig 3)—a phenomenon that was also observed during five successful balance trials in their study. In their data, the furthest COM position behind the heel is around 12.5 cm for one subject, while our simulations show a furthest COM displacement of about 16 cm behind the heel. Consequently, our BRs cover larger areas compared to those observed experimentally, likely due to the experimental design not requiring subjects to fully test the limits of their recovery capacity.

While the current method differs significantly from the torque-driven trajectory optimization method [6], the predicted BRs show a level of comparability with results obtained from optimization [10]. However, it is worth noting that, in general, the predicted BRs using our muscle controller-based method tend to be smaller. This could indicate that the utilization of the MSK model imposes stricter physical and physiological constraints on the feasibility of balance recovery due to limited muscles’ actuation capabilities. The torque-based optimization method does not consider the neural delay and often assumes constant torque capacities during the entire motion, whereas the torque-generation capacity of muscles is affected by muscle states (e.g., fiber length and velocity) and moment arms at different instantaneous states. Our RL framework extends the balance control literature by offering a flexible and robust methodology to identify stability regions and learn balance control strategies through real-time feedback. By integrating muscle dynamics and neural control, our approach provides a more accurate and adaptive model for balance maintenance, addressing the limitations inherent in torque-based methods.

To explore the effects of altered muscle properties on the limits of dynamic balance, we generated RL-based controllers with modified muscle properties, such as maximum isometric fiber forces and activation and deactivation time of the neural excitation-activation delay for all or selected muscles. By analyzing the resulting BRs, we observed that in the first two cases (aging and hemiparesis) the corresponding controllers could still recover from statically balanced states (i.e., COM states with positions within the base of support and zero velocity). Conversely, in the case of hemiplegia, the trained controller could not cover the entire set of statically balanced states. Additionally, we discovered that the hemiplegic model produced a smaller BR, particularly in the region of backward inclination, than that of the case with muscle weakness on both sides. For the case with longer (50%) activation and deactivation time, the BR is much smaller in the region of backward inclination as well. These findings suggest that individuals or patients with muscle weakness or slower neural response times may have difficulties recovering from backward imbalance (e.g., slipping) and are more prone to falling. Similarly, increased neural delay has also been shown to result in narrower stability regions and impaired balance in the elderly and individuals with Parkinson’s Disease [48]. Understanding the effects of varying neural delays on postural stability is crucial for gaining deeper insights into the loss of balance control.

Our numerical experiments demonstrate that these RL-trained muscle controllers have immense potential to study human balance in the neuromuscular domain and provide valuable insights on factors that influence balance improvement or deterioration. The obtained BRs under different muscle conditions offer valuable information regarding the capabilities and limitations of balance recovery of individuals or patients with symptoms such as muscle weakness or hemiplegia. These findings can also be relevant for fall detection and monitoring of the margin of stability during daily activities, especially if a personalized BR can be established through subject-specific modeling and control. Our work can be extended to study balance in other patient populations, such as those with cerebral palsy or Parkinson’s disease. By comparing their predicted BRs against those of healthy subjects and tailoring RL-trained muscle controllers to accommodate specific conditions, we can deepen our understanding of the unique balance challenges encountered by these individuals.

## Conclusion

A novel RL framework was developed to effectively learn muscle-based balance controllers and establish physiologically feasible regions of recoverable balance (i.e., BRs). The keys aspects of this framework include 1) neuromusculoskeletal physics and balance-inspired rewards incorporated in the RL training; 2) dual interlinked neural networks utilized to generate control policies for torques and muscle activations; and 3) a combination of training strategies, including novel RSI, early termination, and CL, employed to test the efficiency and effectiveness of the learning processes. The trained controllers’ performance was evaluated by comparing their outputs under different training regimes. This evaluation involved analyzing the BRs in various muscle conditions and comparing them with the theoretical limits of dynamic balance of the LIP model. This comparison underscored the significance of incorporating human musculoskeletal system’s physiological capabilities and limitations in balance recovery assessments and understanding the impact of muscle deficiencies. This study establishes a foundational approach for stability region-based analysis of human balance, integrating physiological factors and whole-body biomechanics, and advances beyond traditional dynamic balance control and assessment methods.

## Supporting information

S1 File Supporting data. Data for the COM states and trajectories, as well as muscle activations, presented in the figures.
(ZIP)

## References

[1] Levinger P, Dunn J, Bifera N, Butson M, Elias G, Hill KD. High-speed resistance training and balance training for people with knee osteoarthritis to reduce falls risk: study protocol for a pilot randomized controlled trial. Trials. 2017;18(1):384. doi: 10.1186/s13063-017-2129-7 28821271 PMC5563024

[2] Godi M, Franchignoni F, Caligari M, Giordano A, Turcato AM, Nardone A. Comparison of reliability, validity, and responsiveness of the mini-BESTest and Berg Balance Scale in patients with balance disorders. Phys Ther. 2013;93(2):158–67. doi: 10.2522/ptj.20120171 23023812

[3] O’Connor SM, Baweja HS, Goble DJ. Validating the BTrackS Balance Plate as a low cost alternative for the measurement of sway-induced center of pressure. J Biomech. 2016;49(16):4142–5. doi: 10.1016/j.jbiomech.2016.10.020 27789036

[4] Doheny EP, McGrath D, Greene BR, Walsh L, McKeown D, Cunningham C, et al. Displacement of centre of mass during quiet standing assessed using accelerometry in older fallers and non-fallers. Annu Int Conf IEEE Eng Med Biol Soc. 2012;2012:3300–3. doi: 10.1109/EMBC.2012.6346670 23366631

[5] Peng WZ, Mummolo C, Song H, Kim JH. Whole-body balance stability regions for multi-level momentum and stepping strategies. Mechanism and Machine Theory. 2022;174:104880. doi: 10.1016/j.mechmachtheory.2022.104880

[6] Mummolo C, Mangialardi L, Kim JH. Numerical Estimation of Balanced and Falling States for Constrained Legged Systems. J Nonlinear Sci. 2017;27(4):1291–323. doi: 10.1007/s00332-016-9353-2

[7] Koolen T, de Boer T, Rebula J, Goswami A, Pratt J. Capturability-based analysis and control of legged locomotion, Part 1: Theory and application to three simple gait models. The International Journal of Robotics Research. 2012;31(9):1094–113. doi: 10.1177/0278364912452673

[8] Mummolo C, Peng WZ, Gonzalez C, Kim JH. Contact-Dependent Balance Stability of Biped Robots. Journal of Mechanisms and Robotics. 2018;10(2):. doi: 10.1115/1.4038978

[9] Akbas K, Mummolo C. A Computational Framework Towards the Tele-Rehabilitation of Balance Control Skills. Front Robot AI. 2021;8:648485. doi: 10.3389/frobt.2021.648485 34179106 PMC8220374

[10] Mummolo C, Akbas K, Carbone G. State-Space Characterization of Balance Capabilities in Biped Systems with Segmented Feet. Front Robot AI. 2021;8:613038. doi: 10.3389/frobt.2021.613038 33718440 PMC7952635

[11] Tigrini A, Verdini F, Fioretti S, Mengarelli A. Long term correlation and inhomogeneity of the inverted pendulum sway time-series under the intermittent control paradigm. Communications in Nonlinear Science and Numerical Simulation. 2022;108:106198. doi: 10.1016/j.cnsns.2021.106198

[12] Takazawa T, Suzuki Y, Nakamura A, Matsuo R, Morasso P, Nomura T. How the brain can be trained to achieve an intermittent control strategy for stabilizing quiet stance by means of reinforcement learning. Biol Cybern. 2024;118(3–4):229–48. doi: 10.1007/s00422-024-00993-0 38995347 PMC11289178

[13] Winters JM, Stark L. Muscle models: what is gained and what is lost by varying model complexity. Biol Cybern. 1987;55(6):403–20. doi: 10.1007/BF00318375 3567243

[14] Layne CS, Malaya CA, Ravindran AS, John I, Francisco GE, Contreras-Vidal JL. Distinct Kinematic and Neuromuscular Activation Strategies During Quiet Stance and in Response to Postural Perturbations in Healthy Individuals Fitted With and Without a Lower-Limb Exoskeleton. Front Hum Neurosci. 2022;16:942551. doi: 10.3389/fnhum.2022.942551 35911598 PMC9334701

[15] McKay JL, Lang KC, Bong SM, Hackney ME, Factor SA, Ting LH. Abnormal center of mass feedback responses during balance: A potential biomarker of falls in Parkinson’s disease. PLoS One. 2021;16(5):e0252119. doi: 10.1371/journal.pone.0252119 34043678 PMC8158870

[16] Romanato M, Volpe D, Guiotto A, Spolaor F, Sartori M, Sawacha Z. Electromyography-informed modeling for estimating muscle activation and force alterations in Parkinson’s disease. Comput Methods Biomech Biomed Engin. 2022;25(1):14–26. doi: 10.1080/10255842.2021.1925887 33998843

[17] Thelen DG, Anderson FC, Delp SL. Generating dynamic simulations of movement using computed muscle control. J Biomech. 2003;36(3):321–8. doi: 10.1016/s0021-9290(02)00432-3 12594980

[18] Zhou X, Chen X. Design and Evaluation of Torque Compensation Controllers for a Lower Extremity Exoskeleton. J Biomech Eng. 2021;143(1):011007. doi: 10.1115/1.4048572 32975567

[19] Kaminishi K, Jiang P, Chiba R, Takakusaki K, Ota J. Postural control of a musculoskeletal model against multidirectional support surface translations. PLoS One. 2019;14(3):e0212613. doi: 10.1371/journal.pone.0212613 30840650 PMC6402659

[20] Shanbhag J, Wolf A, Wechsler I, Fleischmann S, Winkler J, Leyendecker S, et al. Methods for integrating postural control into biomechanical human simulations: a systematic review. J Neuroeng Rehabil. 2023;20(1):111. doi: 10.1186/s12984-023-01235-3 37605197 PMC10440942

[21] Jones R, Ratnakumar N, Akbaş K, Zhou X. Delayed center of mass feedback in elderly humans leads to greater muscle co-contraction and altered balance strategy under perturbed balance: A predictive musculoskeletal simulation study. PLoS One. 2024;19(5):e0296548. doi: 10.1371/journal.pone.0296548 38787871 PMC11125460

[22] Weng J, Hashemi E, Arami A. Natural Walking With Musculoskeletal Models Using Deep Reinforcement Learning. IEEE Robot Autom Lett. 2021;6(2):4156–62. doi: 10.1109/lra.2021.3067617

[23] Kidziński Ł, Ong C, Mohanty SP, Hicks J, Carroll S, Zhou B, et al., editors. Artificial intelligence for prosthetics: Challenge solutions. The NeurIPS’18 Competition: From Machine Learning to Intelligent Conversations; 2020: Springer.

[24] Luo S, Androwis G, Adamovich S, Su H, Nunez E, Zhou X. Reinforcement Learning and Control of a Lower Extremity Exoskeleton for Squat Assistance. Front Robot AI. 2021;8:702845. doi: 10.3389/frobt.2021.702845 34350214 PMC8326457

[25] Lee S, Park M, Lee K, Lee J. Scalable muscle-actuated human simulation and control. ACM Trans Graph. 2019;38(4):1–13. doi: 10.1145/3306346.3322972

[26] Luo S, Androwis G, Adamovich S, Nunez E, Su H, Zhou X. Robust walking control of a lower limb rehabilitation exoskeleton coupled with a musculoskeletal model via deep reinforcement learning. J Neuroeng Rehabil. 2023;20(1):34. doi: 10.1186/s12984-023-01147-2 36935514 PMC10024861

[27] Joe H-M, Oh J-H. Balance recovery through model predictive control based on capture point dynamics for biped walking robot. Robotics and Autonomous Systems. 2018;105:1–10. doi: 10.1016/j.robot.2018.03.004

[28] Hassanpour H, Mhaskar P, Corbett B. A practically implementable reinforcement learning control approach by leveraging offset-free model predictive control. Computers & Chemical Engineering. 2024;181:108511. doi: 10.1016/j.compchemeng.2023.108511

[29] Hassanpour H, Wang X, Corbett B, Mhaskar P. A practically implementable reinforcement learning‐based process controller design. AIChE Journal. 2023;70(1):. doi: 10.1002/aic.18245

[30] Seth A, Hicks JL, Uchida TK, Habib A, Dembia CL, Dunne JJ, et al. OpenSim: Simulating musculoskeletal dynamics and neuromuscular control to study human and animal movement. PLoS Comput Biol. 2018;14(7):e1006223. doi: 10.1371/journal.pcbi.1006223 30048444 PMC6061994

[31] Lee J, Grey M, Ha S, Kunz T, Jain S, Ye Y, et al. DART: Dynamic Animation and Robotics Toolkit. Journal of Open Source Software. 2018;3(22):.

[32] Millard M, Uchida T, Seth A, Delp SL. Flexing computational muscle: modeling and simulation of musculotendon dynamics. J Biomech Eng. 2013;135(2):021005. doi: 10.1115/1.4023390 23445050 PMC3705831

[33] Todorov E, Erez T, Tassa Y, . Mujoco: A physics engine for model-based control. 2012 IEEE/RSJ International Conference on Intelligent Robots and Systems. 2012.

[34] Zajac FE. Muscle and tendon: properties, models, scaling, and application to biomechanics and motor control. Crit Rev Biomed Eng. 1989;17(4):359–411. 2676342

[35] Hof AL, Gazendam MGJ, Sinke WE. The condition for dynamic stability. J Biomech. 2005;38(1):1–8. doi: 10.1016/j.jbiomech.2004.03.025 15519333

[36] Jie Tan, Liu K, Turk G. Stable proportional-derivative controllers. IEEE Comput Graph Appl. 2011;31(4):34–44. doi: 10.1109/MCG.2011.30 24808157

[37] Schulman J, Wolski F, Dhariwal P, Radford A, Klimov O. Proximal Policy Optimization Algorithms. ArXiv. 2017.

[38] Peng XB, Abbeel P, Levine S, van de Panne M. DeepMimic. ACM Trans Graph. 2018;37(4):1–14. doi: 10.1145/3197517.3201311

[39] Patton JL, Pai Y, Lee WA. Evaluation of a model that determines the stability limits of dynamic balance. Gait Posture. 1999;9(1):38–49. doi: 10.1016/s0966-6362(98)00037-x 10575069

[40] Bengio Y, Louradour J, Collobert R, Weston J, editors. Curriculum learning. Proceedings of the 26th annual international conference on machine learning; 2009.

[41] Robinovitch SN, Heller B, Lui A, Cortez J. Effect of strength and speed of torque development on balance recovery with the ankle strategy. J Neurophysiol. 2002;88(2):613–20. doi: 10.1152/jn.2002.88.2.613 12163514

[42] Simoneau M, Corbeil P. The effect of time to peak ankle torque on balance stability boundary: experimental validation of a biomechanical model. Exp Brain Res. 2005;165(2):217–28. doi: 10.1007/s00221-005-2290-1 15940496

[43] Doherty TJ, Vandervoort AA, Brown WF. Effects of ageing on the motor unit: a brief review. Can J Appl Physiol. 1993;18(4):331–58. doi: 10.1139/h93-029 8275048

[44] Thelen DG. Adjustment of muscle mechanics model parameters to simulate dynamic contractions in older adults. J Biomech Eng. 2003;125(1):70–7. doi: 10.1115/1.1531112 12661198

[45] Mummolo C, Mangialardi L, Kim JH. Quantifying dynamic characteristics of human walking for comprehensive gait cycle. J Biomech Eng. 2013;135(9):91006. doi: 10.1115/1.4024755 23775488

[46] Exarchos I, Jiang Y, Yu W, Karen Liu C. Policy Transfer via Kinematic Domain Randomization and Adaptation. 2021 IEEE International Conference on Robotics and Automation (ICRA)2021. p. 45-51.

[47] Tan J, Zhang T, Coumans E, Iscen A, Bai Y, Hafner D, . Sim-to-Real: Learning agile locomotion for quadruped robots. Robotics: Science and Systems. 2018.

[48] Suzuki Y, Nakamura A, Milosevic M, Nomura K, Tanahashi T, Endo T, et al. Postural instability via a loss of intermittent control in elderly and patients with Parkinson’s disease: A model-based and data-driven approach. Chaos. 2020;30(11):113140. doi: 10.1063/5.0022319 33261318

